# Plant Molecular Phylogenetics and Evolutionary Genomics III

**DOI:** 10.3390/plants15030361

**Published:** 2026-01-24

**Authors:** Aleksey V. Troitsky

**Affiliations:** Belozersky Institute of Physico-Chemical Biology, Lomonosov Moscow State University, 119992 Moscow, Russia; bobr@belozersky.msu.ru

Comparative analyses of the structure of genes and genomes are an important tool in elucidating the phylogenetic relationships of organisms, the evolutionary pathways of genetic material, and its structural and functional basis. The evolutionary paradigm serves as a blueprint for investigations in almost all biological disciplines. Over time, the number of publications on this topic has only increased (Figure 1).

A PubMed search (https://pubmed.ncbi.nlm.nih.gov, accessed on 11 December 2025) identified more than 9000 articles on Plant Molecular Phylogenetics published in 2024–2025. The publications presented in this Special Issue represent a fairly representative cross-section of research in the topic.

The objects of analysis in about half of the articles were the genomes of chloroplasts (CP) from representatives of five families of angiosperms, as well as Sphenopsida. By comparing the CP genomes of 48 accessions of varieties and species of *Zea*, Montenegro et al. [1] established the polyphyletic origin of Peruvian purple maize in South America and the complex circumstances of its domestication with recurrent gene flow from wild relatives.

Lei et al. [2] reconstructed the evolutionary relationships of *Kitagawia* (Apiaceae) species, which are poorly understood, using CP genomes and nuclear rDNA sequences. The phylogenetic trees constructed from CDS of 27 CP genomes and nuclear ITS + ETS from the rDNA of *Kitagawia* are not completely congruent, as is commonly found in other studies, but indicate that all six examined *Kitagawia* species were divided into Subclade I and Subclade II within the tribe Selineae, and they were all distant from the representative members of *Peucedanum* sensu stricto. This study supported the separation of *Kitagawia* from *Peucedanum* sensu lato, confirming that *Kitagawia* belongs to Selineae, and that two species (*K. praeruptora* and *K. formosana*) within Subclade II should be placed in a new genus.

Goryunova et al. [3] sequenced the complete chloroplast genomes of Solanum tuberosum accessions for the first time, with five out of the six major cytoplasmic genome types. Based on the phylogenetic analysis of the complete plastome sequences, five main evolutionary branches of CP genomes (clades A–E) can be distinguished within the Petota section. Clade D comprises accessions with the M-, A, and P cytoplasm types. Samples with A- and P- cytoplasm formed isolated distant groups within a large and polymorphic group of samples with M-type cytoplasm, suggesting that the A and P genomes arose independently. Moreover, given the independent origin of the A- and P-type cytoplasmic groups, it seems unlikely that Andean cultivated tetraploids (*S. tuberosum* group Andigena) evolved directly from early landrace diploids (*S. tuberosum* groups Stenotomum and Phureja) through autopolyploidy. Accessions with the sterilizing cytoplasm types W, D, and T are part of the E clade. The divergence time between the D and E clades can be estimated as 4.71 MYA (3.78–5.70). The M type of cytoplasm appears to be the most ancient. The findings suggest that the diversity of the T-genome in *S. tuberosum* Group Tuberosum could be initially low due to the bottleneck already occurring at the origin of the Chilean clade. Revealed variations in the rbcL gene sequence may be one of the factors causing differences in the appearance of economically important traits between species with A and T-type cytoplasm.

Comparative analyses of 45 *Plantago* species (Plantaginaceae) based on fully sequenced plastomes identified the most variable mutational hotspots that were not suitable for the development of species-specific molecular markers; however, species-specific polymorphisms could discriminate *P. lanceolata* from its closest relatives [4]. Molecular evolutionary analyses indicated that eleven protein-coding genes involved in different functions in *Plantago* plastomes underwent positive selection, suggesting they might have contributed to enhancing species’ adaptation during the evolutionary history of *Plantago*. It is possible that the complex climatic oscillations that took place in the late Miocene and the beginning of the Pliocene could have at least in part contributed to determining the selective patterns observed in *rpo*B and *rpo*C2 genes of the studied *Plantago*.

Samigullin et al. [5] sequenced plastomes of 16 species from the tribe Loteae (Fabaceae) and reconstructed the phylogeny of this tribe basing on 23 complete CP genomes. Comparative analysis revealed several regions (*pet*N-*trn*C and *rps*16-*acc*D spacers from the LSC region of CP DNA, and in the *ycf* 1 gene within the SSC) as the most variable and potentially useful for phylogenetic purposes.

For horsetails (Sphenopsida), Satjara et al. sequenced the complete CP genome of *Equisetum xylochaetum* [6] but used only five markers (*atp*B, *mat*K, *rpo*B, *rps*4, and *trn*L-F) to reconstruct the phylogeny of four species. In addition to this, the TCS haplotype networks of *atp*B, *mat*K, *rpo*B, *rps*4, and *trn*L-F were calculated for 105 *Equisetum* accessions. Some *Equisetum* species were categorized as more than a single haplotype.

Of the nuclear genes, rDNA is the most frequently used in molecular phylogenetics. Internal transcribed spacers (ITS) of nuclear rDNA were used as barcodes for the identification of medicinal plants from the Tianshan wild fruit forest region [7] and to study the intragenomic polymorphism of cultivated and wild *Avena* species [8]. According to the data obtained, the diploid *A. strigosa* could have evolved independently of the polyploid-cultivated species, and the tetraploid *A. abyssinica* could be a cultivated derivative of *A. vaviloviana* and hexaploid-cultivated species, *A. byzantina* and *A. sativa*, which could have a different origin. *A. sativa* could be the cultivated form of *A. fatua*, whereas *A. byzantina* could originate independently.

Using RAD-seq technology, 2996 high-quality single-nucleotide polymorphisms (SNPs) were identified in *Stewartia gemmata* and *S. acutisepala* (Theaceae), the analysis of which was used to infer the evolutionary relationships of two species [9]. The results suggested strong monophyly of both species, and *S. acutisepala* was nested within *S. gemmate*.

Structure, evolution, and expression of several gene families were studied. The objects of the research were the lipoxygenase gene families (*LOX*) in angiosperms [10], the aquaporin (*AQP*) family in papaya *Carica papaya* (Brassicales) [11], the *MYB* gene family in *Chrysanthemum X morifolium* (Asteraceae) [12], and TCP Interactor containing EAR motif proteins genes (*TIE*) in Embryophyta [13]. Evolutionary trees were constructed for 247 *LOX* genes from 23 species of angiosperms and basal plants, 550 *AQP* genes from seven species, 1945 *MYB* genes in six plant species, and 102 TIE proteins from 33 embryophytes. Henning et al. [14], analyzing the newly sequenced and annotated draft genome of the *Turnera subulata* (Passifloraceae), identified in this plant 169 candidate genes of the three families *BAHD*, *SPH*, and *YUC* and reconstructed evolutionary trees for 1741 S-families members from 57 embryophytes.

Two articles are reviews. The first is devoted to the role of transposons in the evolution of polyploid plant genomes [15]. Plant transposons are the most variable part of the genome, causing extensive changes in the overall structure and gene function of the genome, affecting the genome regulatory network and evolution. Transposons and polyploidy are interconnected because polyploidy can induce transposon activity, and transposons enable polyploidy to produce new variants. The authors formulate key questions about the role of transposons in polyploid plant genome evolution and their regulatory mechanisms, which deserve further study. The second review [16] summarizes current knowledge on the biodiversity, taxonomy, and physiology of the genus *Ulva* and assesses the applications of these algae in food, feed, bioremediation, biofuels, pharmaceuticals, and biomaterials.

## Figures and Tables

**Figure 1:**
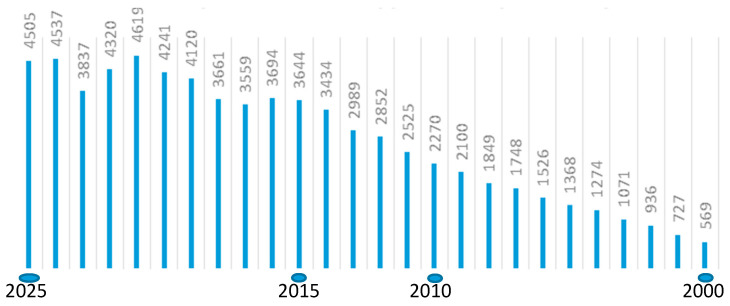
PubMed search query: (molecular) AND (phylogeny) OR (phylogenetic) AND (plants) NOT (pathogen) NOT (virus), 2000–2025.
